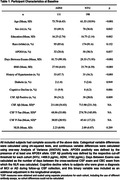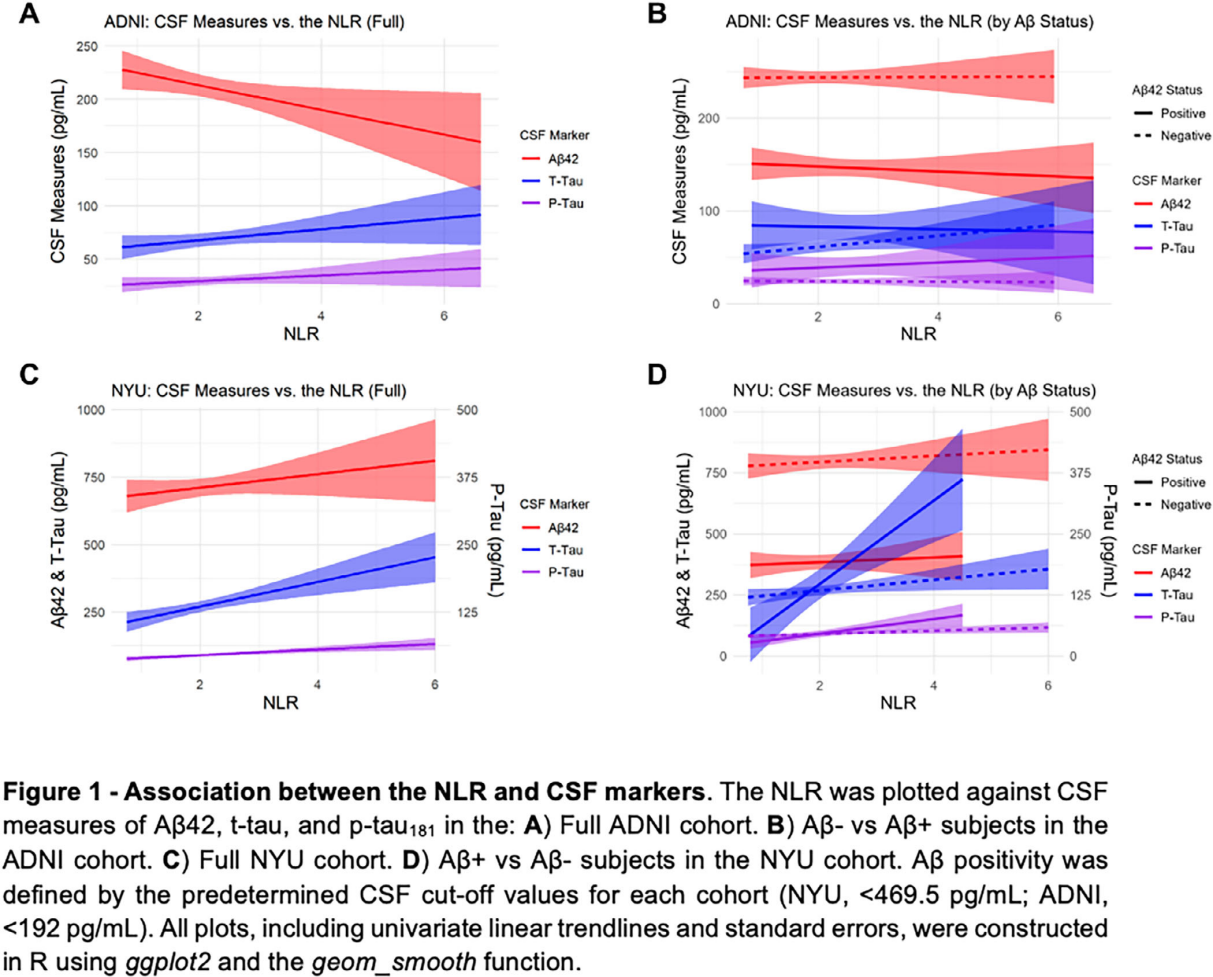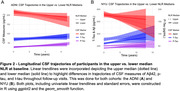# The neutrophil to lymphocyte ratio associates with markers of Alzheimer’s disease pathology in cognitively unimpaired elderly people

**DOI:** 10.1002/alz.092957

**Published:** 2025-01-03

**Authors:** Tovia Jacobs, Sean R Jacobson, Juan Fortea, Jeffrey S Berger, Alok Vedvyas, Karyn Marsh, Moses Gonzalez, Luisa F Figueredo, Chelsea Reichert Plaska, Esther M Blessing, Rebecca A Betensky, Henry Rusinek, Lidia Glodzik, Thomas Wisniewski, Ricardo S. Osorio, Mony J. de Leon, Jaime Ramos Cejudo, Alzheimer’s Disease Neuroimaging Initiative (ADNI)

**Affiliations:** ^1^ NYU Grossman School of Medicine, New York, NY USA; ^2^ New York University (NYU) Grossman School of Medicine, New York, NY USA; ^3^ Sant Pau Memory Unit, Hospital de la Santa Creu i Sant Pau, Biomedical Research Institute Sant Pau, Universitat Autònoma de Barcelona, Barcelona Spain; ^4^ New York University School of Medicine, New York, NY USA; ^5^ New York University, New york city, NY USA; ^6^ NYU Alzheimer’s Disease Research Center, New York, NY USA; ^7^ Brain Health Imaging Institute, Department of Radiology, Weill Cornell Medicine, New York, NY USA; ^8^ Brain Health Imaging Institute, Department of Radiology, Weill Cornell Medicine, New York City, NY USA; ^9^ Laboratory of Neuro Imaging (LONI), University of Southern California, Los Angeles, CA USA

## Abstract

**Background:**

An elevated neutrophil‐lymphocyte ratio (NLR) has been associated with Alzheimer’s disease (AD). However, an elevated NLR has also been implicated in many other conditions that are risk factors for AD, prompting investigation into whether the NLR is directly linked with AD pathology or a result of underlying comorbidities.

**Method:**

We explored the relationship between the NLR and AD biomarkers in the cerebrospinal fluid (CSF) of cognitively unimpaired (CU) subjects. Adjusting for sociodemographics, APOE4, and common comorbidities, we investigated these associations in two cohorts: the Alzheimer’s Disease Neuroimaging Initiative (ADNI) and NYU Center for Brain Health (CBH). Specifically, we examined associations between the NLR and cross‐sectional measures of amyloid‐β42 (Aβ42), total tau (t‐tau), and phosphorylated tau_181_ (p‐tau_181_), as well as the trajectories of these CSF measures obtained longitudinally.

**Result:**

A total of 111 ADNI and 190 NYU participants classified as CU with available NLR, CSF, and covariate data were included. Compared to NYU, ADNI participants were older (73.79 vs. 61.53, p<0.001), had a higher proportion of males (49.5% vs. 36.8%, p = 0.042), higher BMIs (27.94 vs. 25.79, p<0.001), higher prevalence of hypertensive history (47.7% vs. 16.3%, p<0.001), and a greater percentage of Aβ‐positivity (34.2% vs. 20.0%, p = 0.009) (**Table 1**). In the ADNI cohort, we found cross‐sectional associations between the NLR and CSF Aβ42 (β = ‐12.193, p = 0.021), but not t‐tau or p‐tau_181_. In the NYU cohort, we found cross‐sectional associations between the NLR and CSF t‐tau (β = 26.812, p = 0.019) and p‐tau_181_ (β = 3.441, p<0.001), but not Aβ42. In the NYU cohort alone, subjects classified as Aβ+ (n = 38) displayed a stronger association between the NLR and t‐tau (β = 100.476, p = 0.037) compared to Aβ‐ subjects or the non‐stratified cohort (**Figure 1**). In both cohorts, the same associations observed in the cross‐sectional analyses were observed after incoporating longitudinal CSF data (**Figure 2**).

**Conclusion:**

We report associations between the NLR and Aβ42 in the older ADNI cohort, and between the NLR and t‐tau and p‐tau_181_ in the younger NYU cohort. Associations persisted after adjusting for comorbidities, suggesting a direct link between the NLR and AD. However, changes in associations between the NLR and specific AD‐biomarkers may occur as part of immunosenescence.